# Supplementary material for: From a genome-wide screen of RNAi molecules against SARS-CoV-2 to a validated broad-spectrum and potent prophylaxis
Source: Commun Biol. 2023 Mar 16;6:277. doi: 10.1038/s42003-023-04589-5 (PMC10019795; doi:10.1038/s42003-023-04589-5)
Supplement: Supplementary file 2 — SUPPLEMENTAL MATERIAL [file 42003_2023_4589_MOESM2_ESM.pdf]

Supplementary Material

Supplementary Figures

Supplementary Figure 1

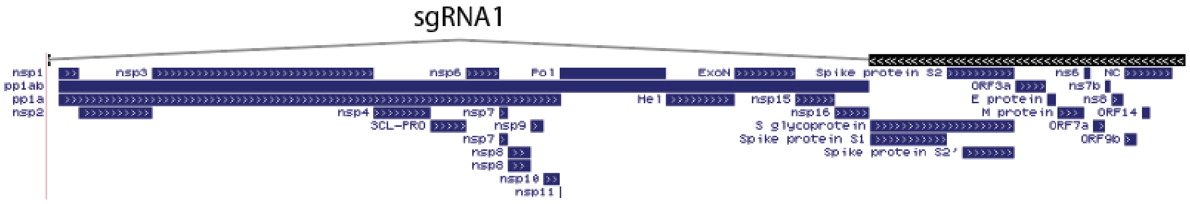

Supplementary Figure 1: Scheme of subgenomic RNA1. The negative strand subgenomic RNA1 (black) includes all the structural proteins and the leader peptide. Its 5' position starts from the right most region. In blue: the various proteins in the SARS-CoV-2 genome.

Supplementary Figure 2

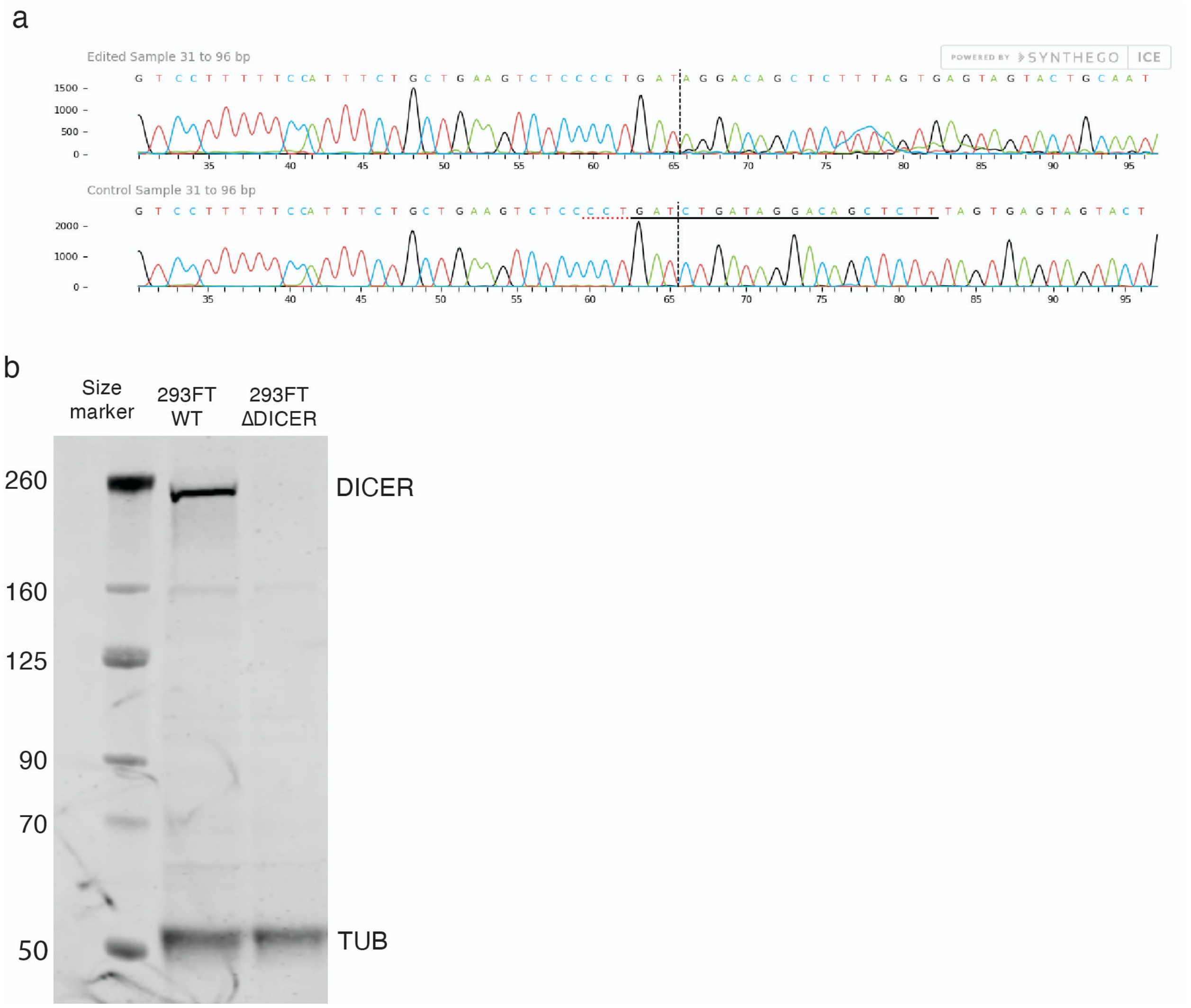

Supplementary Figure 2: Validation of DICER KO in 293FT cells. (a) Sanger sequencing analysis of the gRNA region was performed using the Synthego ICE tool shows a 5 nucleotides deletion; **(b)** Western blot analysis of the parental and KO cells shows complete deletion of DICER protein in the KO cells.

Supplementary Figure 3

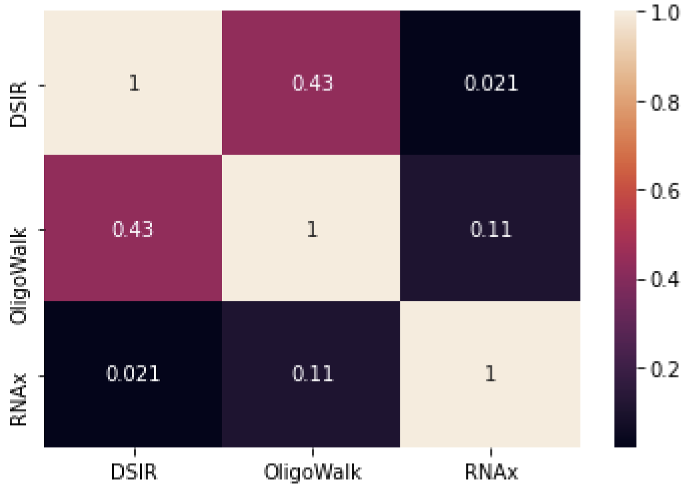

Supplementary Figure 3: The cross-correlation matrix between DSIR, Oligowalk, and RNAx with respect to the sorted siRNA candidate list.

Supplementary Figure 4

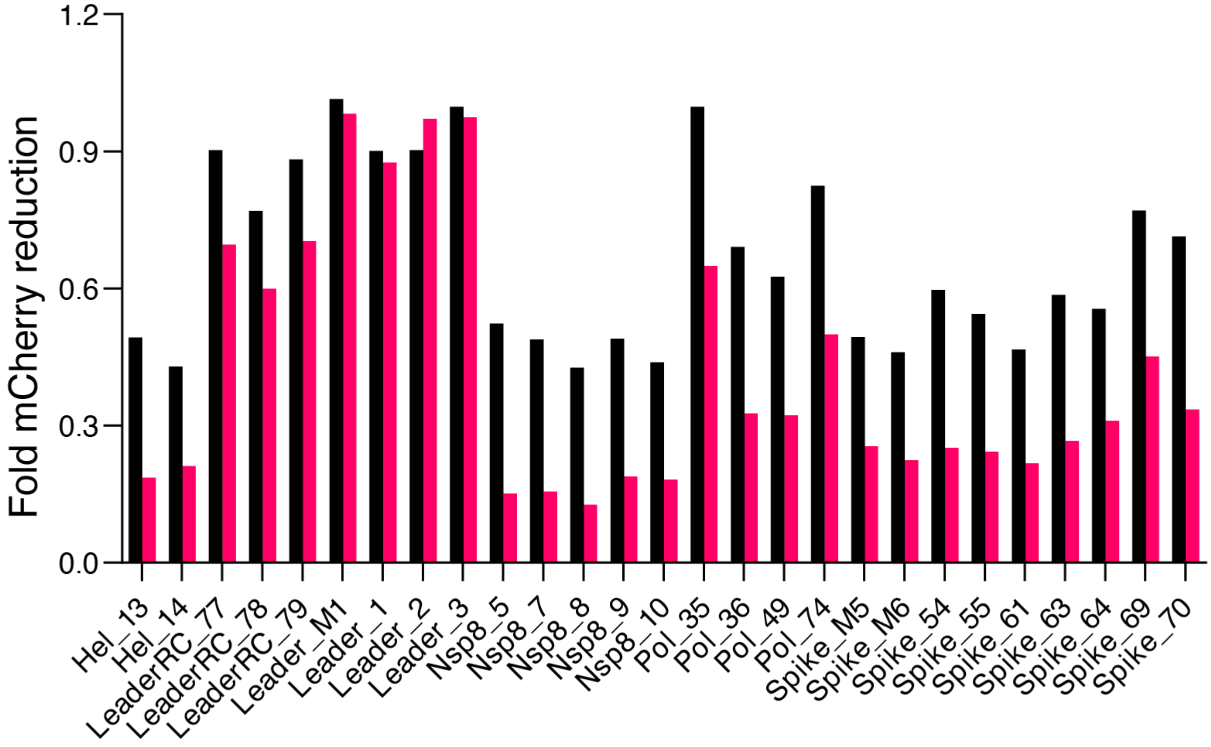

Supplementary Figure 4: Validation of the selected siRNAs by a reporter assay. Cells were treated with each siRNA at 100pM (black) or 500pM (pink) together with their respective target site fused to the 3'UTR of mCherry. siRNA activity was calculated by measuring the %mCherry positive signal in the GFP positive cell population.

Supplementary Figure 5

a

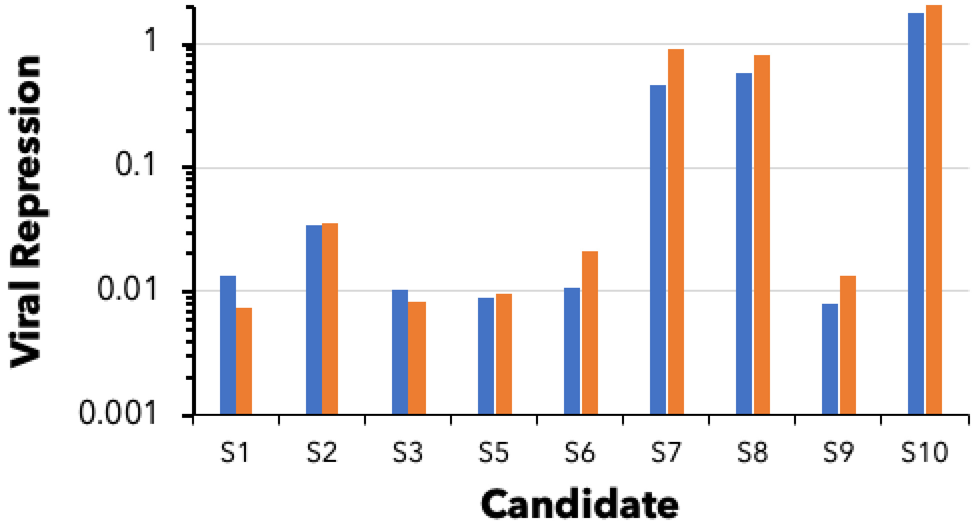

b

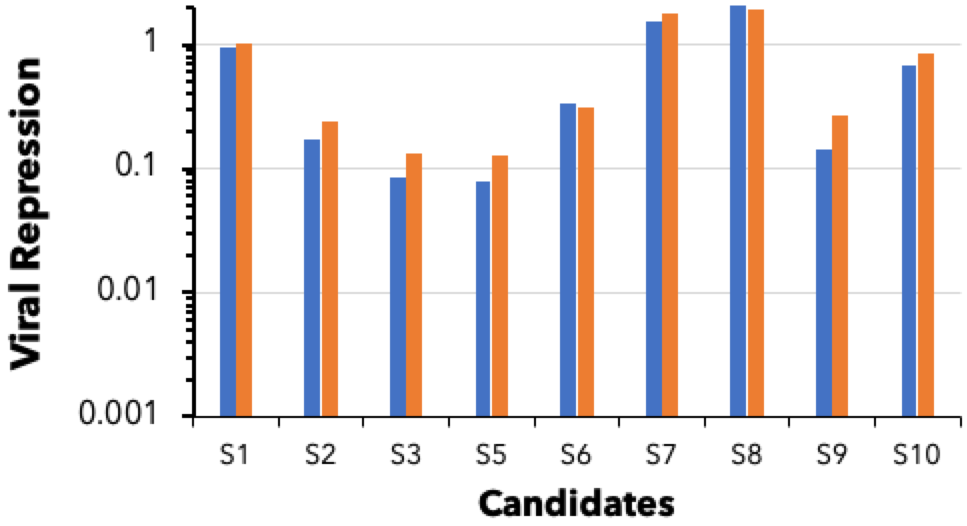

Supplementary Figure 5: Viral load repressions after siRNA prophylaxis in VeroE6 cells as measured by qPCR. The level is calibrated to 100% using siRNA against GFP. Blue: RdRP; Orange: E-gene; **(a) Challenging the cells with 6000x$TCID_{50}$ (b) Challenging the cells with 60x$TCID_{50}$**.

Supplementary Figure 6

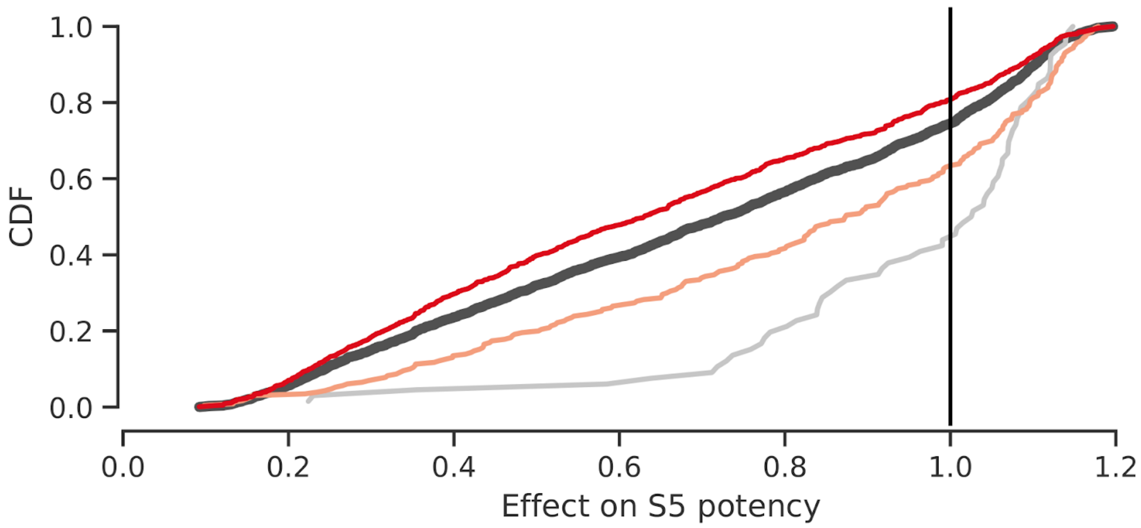

Supplementary Figure 6. The cumulative distribution function of the effect of mutations on S5. The vertical lines signifies no difference than the screen score of a target site without any mutation. Black: the distribution of effects of all 2143 single and double mutations. Grey: the distribution of all single mutations. Orange: the distribution of all double transition mutations. Red: the distribution of all double transversion mutations.

Supplementary Figure 7

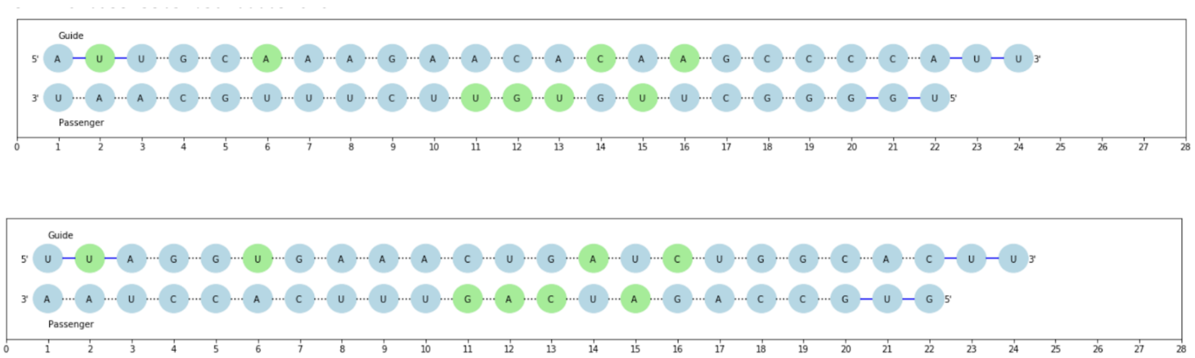

Supplementary Figure 7. Chemical modifications of in-vivo tested siRNAs. Hel14 (top) and S5 (bottom). Blue: 2'-OMe; Green: 2'-F, dotted line: phosphate; solid line: phosphorothioate.

Supplementary Figure 8

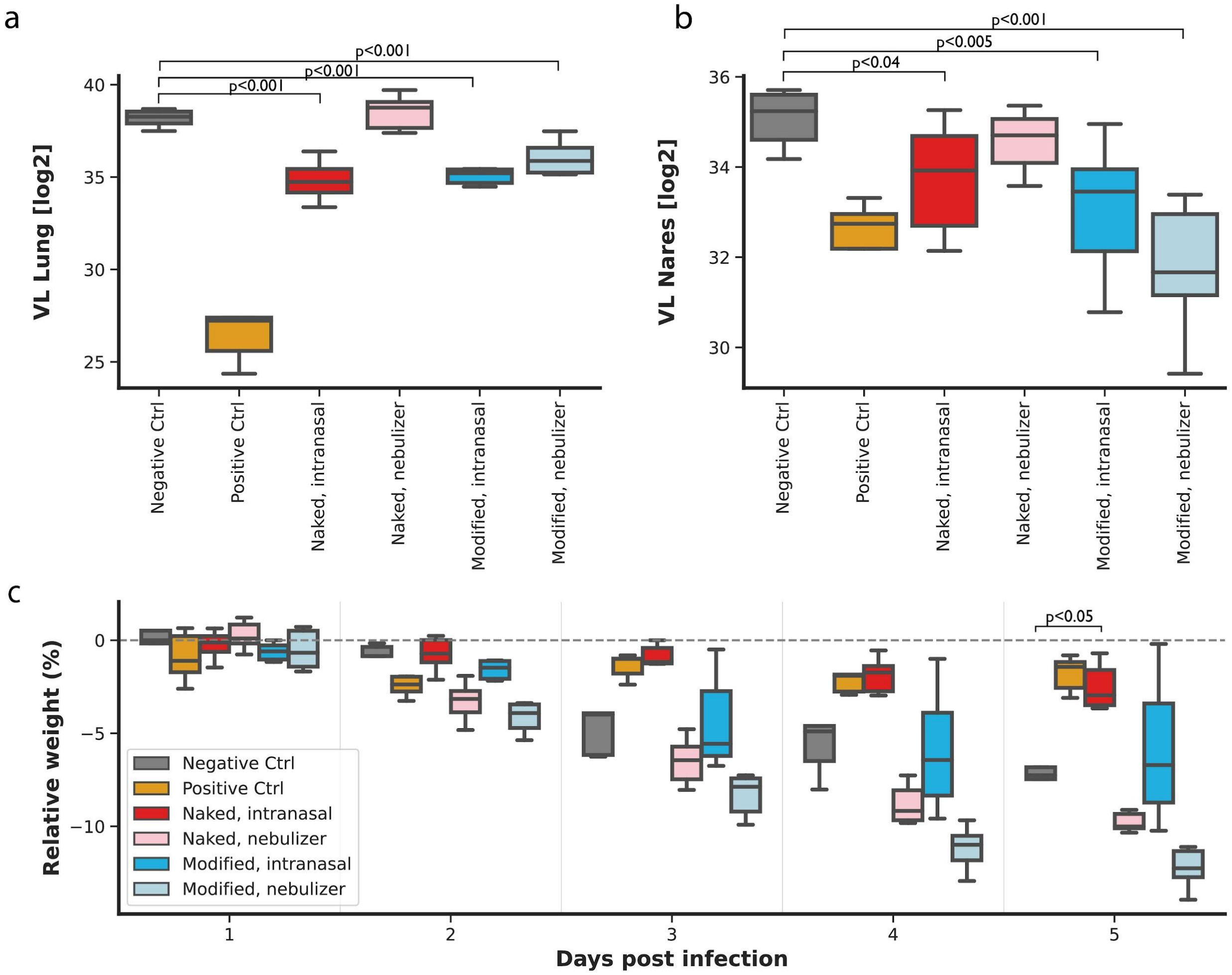

Supplementary Figure 8. Prophylactic treatments of SARS-CoV-2 infection in Syrian hamsters under various formulations. Syrian hamsters (n=6 per group) pre-treated with a non-targeting siRNA (negative control; grey), the LY-CoV555 antibody (positive control; orange) or our lead siRNA cocktail in four different formulations, all of which containing our delivery moiety: naked siRNAs intranasally (red), nebulized naked siRNAs (pink), modified siRNA intranasally (blue), nebulized modified siRNA (light blue) **(a-b) Viral load (VL) by qPCR.** All measurements are based on qPCR of the RdRP gene five days post infection from either homogenised; **(c) Weight change after infection**. The box plot presents the change in weight by treatment group relative to the infection day. All p-values are adjusted to multiple hypotheses of the four treatment arms and are based on a parametric bootstrapping as described in **Methods**. Horizontal lines represent the median, box edges represent the 25% and 75% quartiles, and the whiskers represent the furthest data points within up to 50% of the interquartile range

Supplementary Figure 9

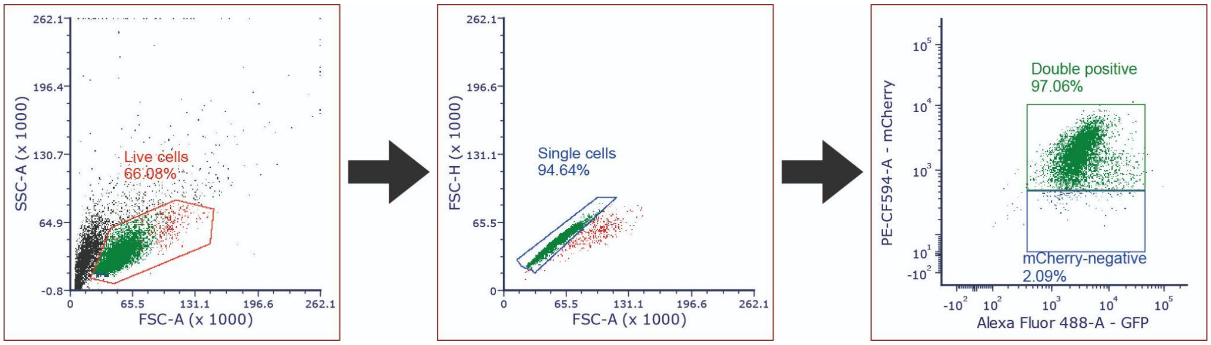

Supplementary Figure 9. Flow cytometry gating strategy. To analyse siRNA activity using the reporter cell line, we measured the percentage of $GFP^+/mCherry^-$ cells within the live single cell population.

Supplementary Table 1

**Condition**	**# of siRNA targets on the SARS-CoV-2 plus strand genome**	**# of siRNA targets on the negative strand subgenomic RNA1 of SARS-Cov-2**
Initial number of targets	29,881	8,384
… and no restriction sites for EcoRI, MluI, XhoI, and MfeI1[(1)]	28,403	7,735
… … and no sites with homopolymers of >4nt across the oligo[(2)]	23,162	5,979
… … … and between nine to eighteen A/U nucleotides in guide strands[(3)]	22,870	5,850
… … … … and first guide position is A or U[(3)]	14,024	3,493
… … … … … and no matches to the human transcriptome[(4)] (match = up to 1nt difference in the seed region or up to 2nt difference in the entire guide to a human transcript)	13,193	3,278
**Total passed**	**16,471**	

Supplementary Table 1: The filtering steps for initial selection of siRNA targets. (1) The restriction enzymes in our cloning strategy. Oligos with these sites will be cut in the middle and therefore are unclonable (2) previous studies[18] have shown that long homopolymers above 4nt are likely to introduce synthesis, PCR, and sequencing errors. Therefore, we excluded such sites (3) Previous work[13] has identified these rules of thumb as correlating with potent shRNA response (4) We selected these rules to reduce the likelihood of off-target effects to the human transcriptome.

Supplementary Table 2

Name	IC50-1	IC50-1 s.e.	IC50-2	IC50-2 s.e.	IC50-both	IC50-both s.e.
**S1**	41.1	5.0	35.4	3.4	38.9	3.8
**S2**	17.3	1.1	14.4	0.8	16.0	0.9
**S3**	10.1	0.5	11.5	0.6	10.7	0.6
**S4**	17.6	1.3	15.5	0.7	16.6	0.7
**S5**	27.9	1.9	27.9	2.0	27.9	2.0
**S6**	49.4	2.9	37.5	2.0	42.6	1.9
**S7**	254.9	52.0	264.5	44.7	259.7	45.4
**S8**	9.4	0.4	8.0	0.3	8.6	0.3
**S9**	1291.5	79.4	1703.1	77.9	1469.5	73.5
**S10**	21.9	1.7	17.0	1.4	19.3	1.4

Supplementary Table 2: Replicate-specific IC50 estimates of the siRNA candidates. For each siRNA candidate, we report its IC50 estimate (and its standard error) in replicate 1 (IC50-1), in replicate 2 (IC50-2), and in the analysis that considers both replicates (IC50-both).

Supplementary Table 3

Name	Gene	Guide
NSP-8	NSP8	UAAGAACAACUUCAGAAUC
Hel14	Hel	AUUGCAAAGAACACAAGCC
Pol49	RDRP	UUUCUUGGAAGCGACAACA
Spike 61	Spike	UAUCAAACCUCUUAGUACC
Spike M6	Spike	UUAUGUUAGACUUCUCAGUG

Supplementary Table 3: The tested guides with a live virus from the open source discovery method.
