# Supplementary material for: From a genome-wide screen of RNAi molecules against SARS-CoV-2 to a validated broad-spectrum and potent prophylaxis
Source: Commun Biol. 2023 Mar 16;6:277. doi: 10.1038/s42003-023-04589-5 (PMC10019795; doi:10.1038/s42003-023-04589-5)
Supplement: Supplementary file 3 — Description of Additional Supplementary Files [file 42003_2023_4589_MOESM3_ESM.pdf]

Description of Additional Supplementary Files

File name: Supplementary Data 1
Description: Data set for Figure S4

File name: Supplementary Data 2
Description: Data set for Figure S5

File name: Supplementary Data 3
Description: Data set for Figure S8a

File name: Supplementary Data 4
Description: Data set for Figure S8b

File name: Supplementary Data 5
Description: Data set for Figure S8c
